# Supplementary material for: ANCA: artificial nucleic acid circuit with argonaute protein for one-step isothermal detection of antibiotic-resistant bacteria
Source: Nat Commun. 2023 Dec 5;14:8033. doi: 10.1038/s41467-023-43899-4 (PMC10697997; doi:10.1038/s41467-023-43899-4)
Supplement: Supplementary file 1 — Supplementary Information [file 41467_2023_43899_MOESM1_ESM.pdf]

Supplementary Information

ANCA: Artificial nucleic acid circuit with argonaute protein for one-step isothermal detection of antibiotic-resistant bacteria

Hyowon Jang[1], Jayeon Song[1,2,3], Sunjoo Kim[4], Jung-Hyun Byun[4], Kyoung G. Lee[5], Kwang-Hyun Park[6], Euijeon Woo[6,7], Eun-Kyung Lim[1,8,9], Juyeon Jung[1,9], and Taejoon Kang[1,9*]

[1]Bionanotechnology Research Center, Korea Research Institute of Bioscience and Biotechnology (KRIBB), 125 Gwahak-ro, Yuseong-gu, Daejeon 34141, Republic of Korea

[2]Center for Systems Biology, Massachusetts General Hospital Research Institute, 175 Cambridge Street, Boston, MA 02114, USA

[3]Department of Radiology, Massachusetts General Hospital, Harvard Medical School, 55 Fruit Street, Boston, MA 02114, USA

[4]Department of Laboratory Medicine, Gyeongsang National University Hospital, Gyeongsang National University College of Medicine, 79 Gangnam-ro, Jinju-si, Gyeongsangnam-do 52727, Republic of Korea

[5]Division of Nano-Bio Sensors/Chips Development, National NanoFab Center (NNFC), 291 Daehak-ro, Yuseong-gu, Daejeon 34141, Republic of Korea

[6]Disease Target Structure Research Center, KRIBB, 125 Gwahak-ro, Yuseong-gu, Daejeon 34141, Republic of Korea

[7]Department of Biomolecular Science, KRIBB School of Biotechnology, University of Science and Technology (UST), 217 Gajeong-ro, Yuseong-gu, Daejeon 34113, Republic of Korea

[8]Department of Nanobiotechnology, KRIBB School of Biotechnology, UST, 217 Gajeong-ro, Yuseong-gu, Daejeon 34113, Republic of Korea

[9]School of Pharmacy, Sungkyunkwan University (SKKU), 2066 Seobu-ro, Jangan-gu, Suwon-si, Gyeongi-do 16419, Republic of Korea

[*]E-mail : kangtaejoon@gmail.com

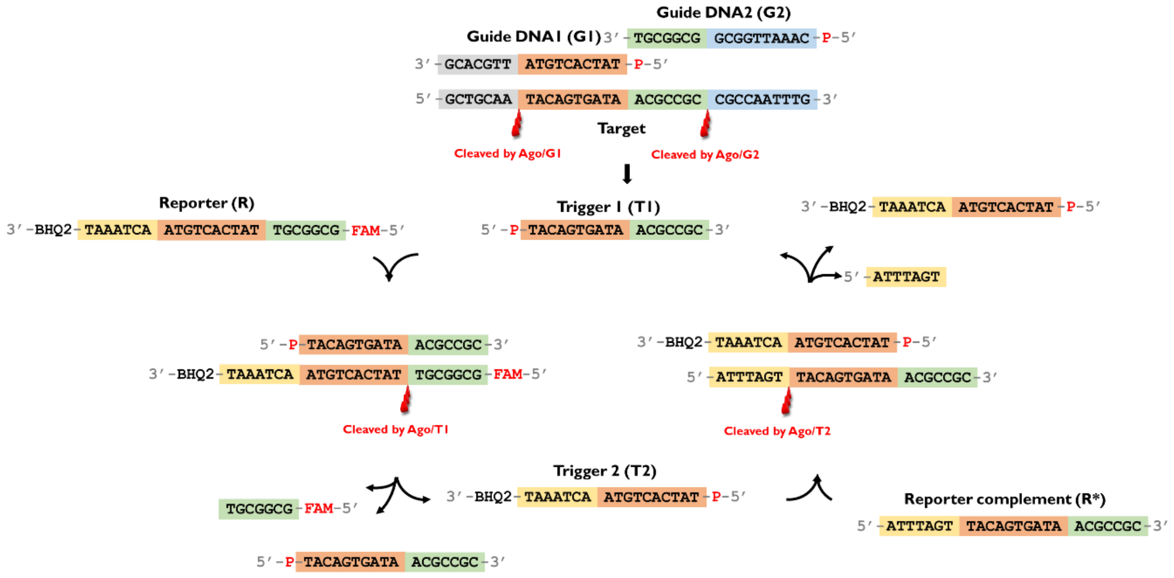

Supplementary Fig. 1 Sequence-based illustration of KPC circuit.

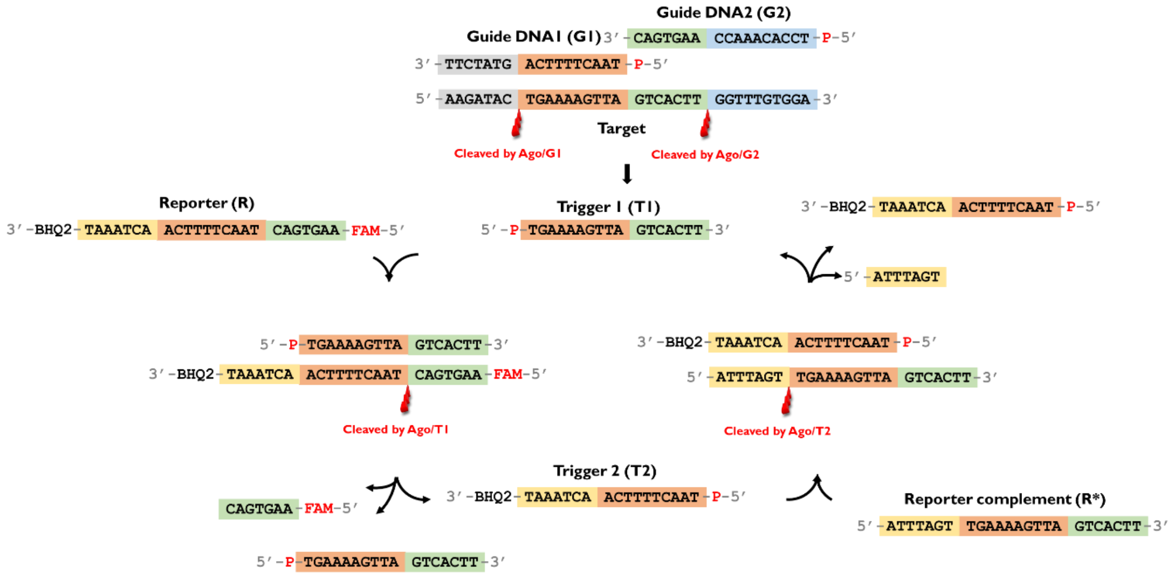

Supplementary Fig. 2 Sequence-based illustration of IMP circuit.

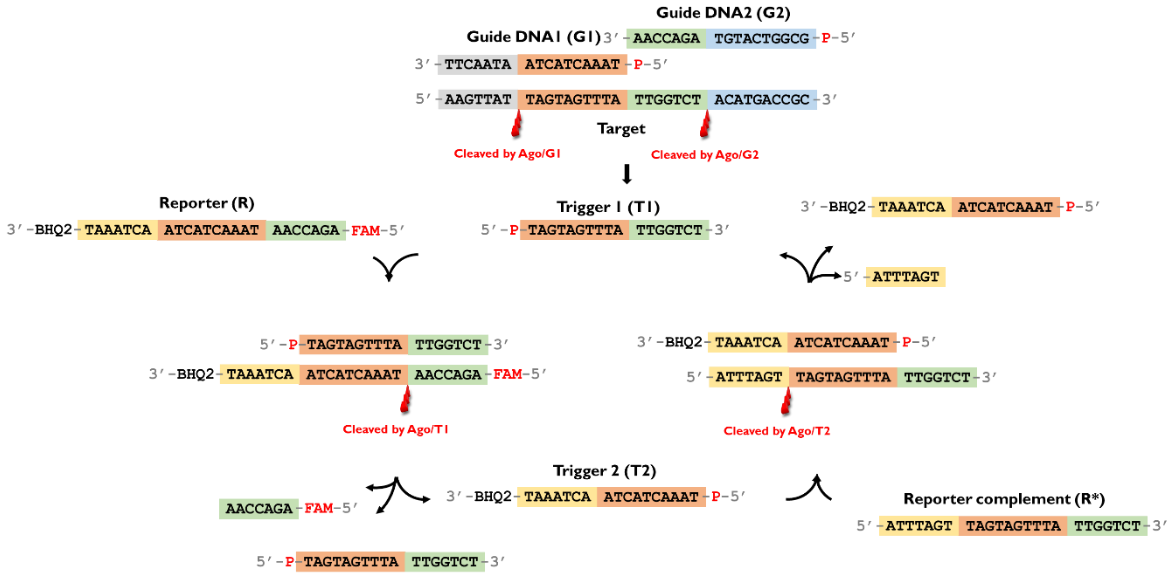

Supplementary Fig. 3 Sequence-based illustration of VIM circuit.

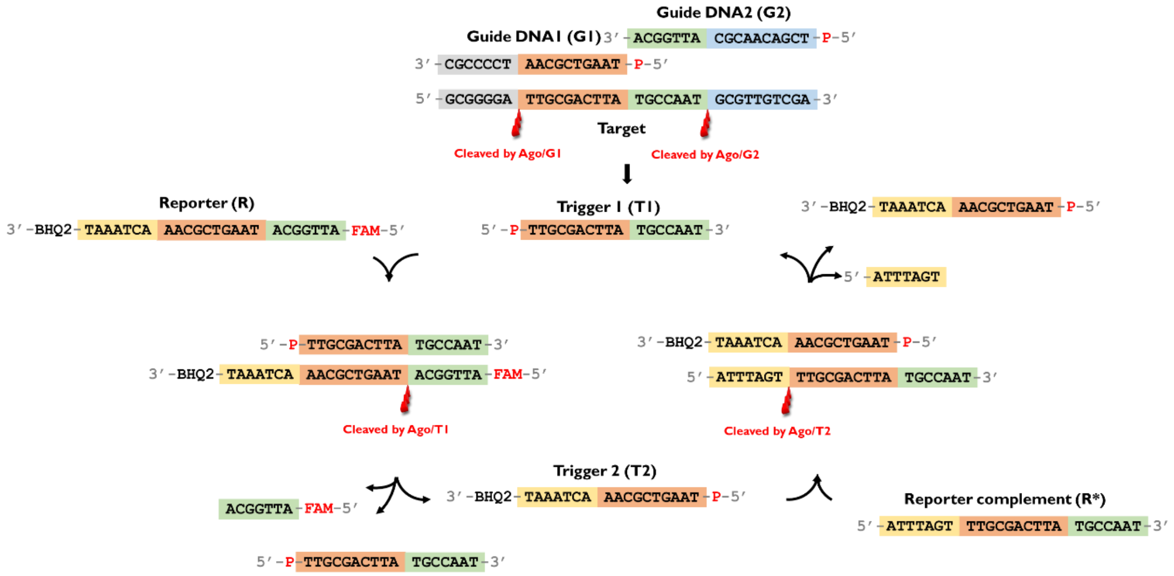

Supplementary Fig. 4 Sequence-based illustration of NDM circuit.

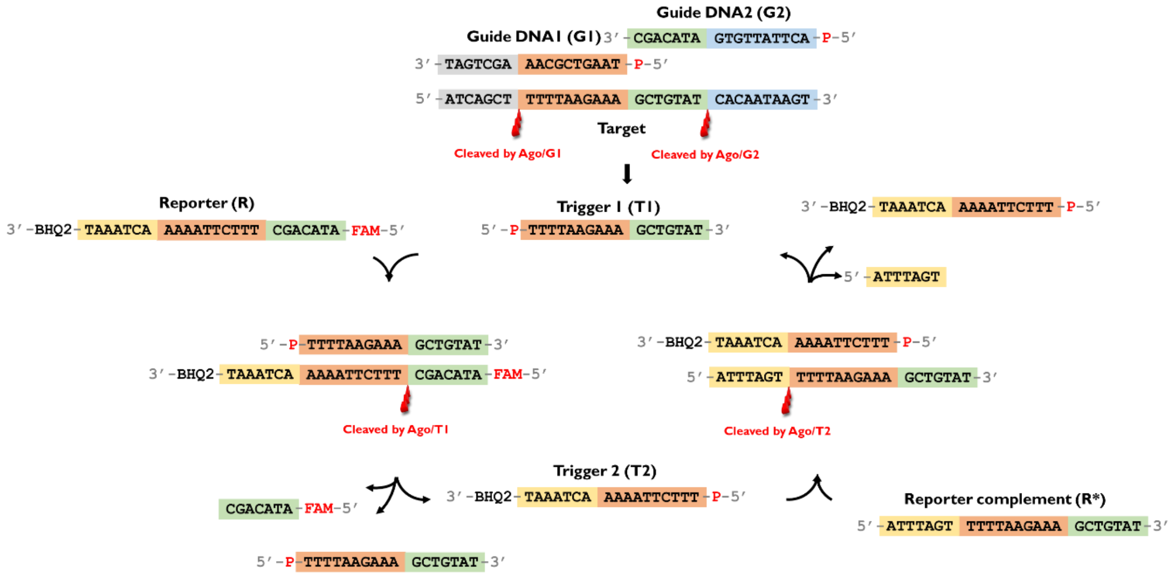

Supplementary Fig. 5 Sequence-based illustration of OXA-48 circuit.

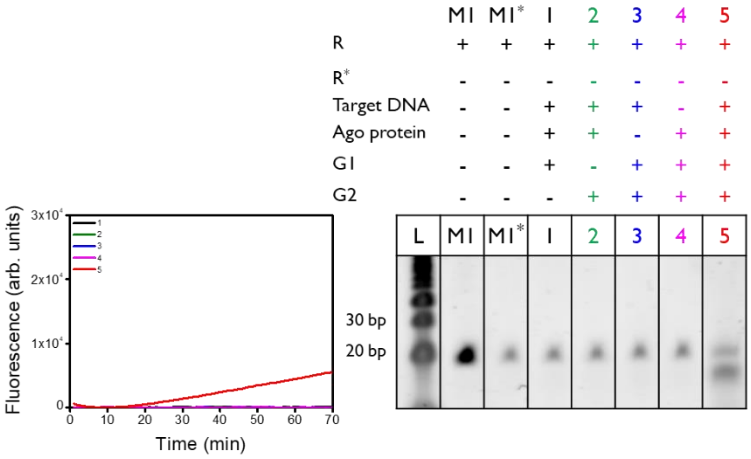

Supplementary Fig. 6 Time-dependent fluorescence intensities during ANCA reaction without R* (left) and corresponding PAGE analysis result (right) under various components. [Target DNA] = 10 nM, [R] = 500 nM or 1000 nM (only M1), [G1] = 25 nM, [G2] = 25 nM, [Ago] = 200 nM, [$MgCl_2$] = 10 mM, [NaCl] = 75 mM, and [BSA] = 10 μg/mL. The experiments were independently replicated three times.

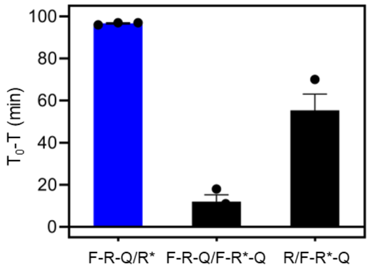

Supplementary Fig. 7 Plot of T_0 – T value by varying R and R* for ANCA method (n = 3 independent experiments, error bar = standard deviation). When fluorophore and quencher were attached to only R sequence (F-R-Q/R*), the highest T_0 – T value was obtained. T_0 represents the threshold time (reaction time at which the fluorescence intensity reached 10,000) for negative sample, and T denotes the threshold time for positive sample. [Target DNA] = 10 nM, [R or F-R-Q] = 500 nM, [R* or F-R*-Q] = 500 nM, [G1] = 25 nM, [G2] = 25 nM, [Ago] = 200 nM, [$MgCl_2$] = 10 mM, [NaCl] = 75 mM, and [BSA] = 10 μg/mL. Data are presented as mean values +/- standard deviation.

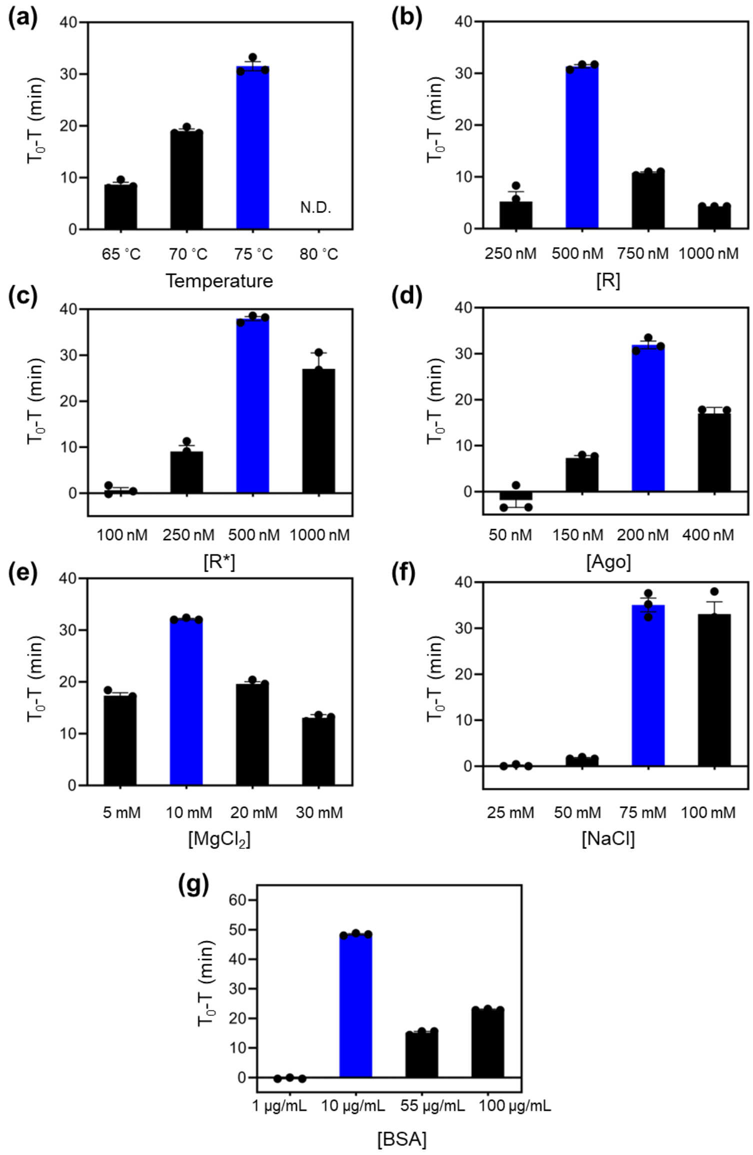

Supplementary Fig. 8 (a-g) Plot of T_0 – T value by varying (a) reaction temperature and concentrations of (b) R, (c) R*, (d) Ago protein, (e) $MgCl_2$, (f) NaCl, and (g) BSA for ANCA method (n = 3 independent experiments, error bar = standard deviation, N.D. = threshold time was not determined). When the reaction temperature was 75 °C and the concentrations of R, R*, Ago protein, $MgCl_2$, NaCl, and BSA were 500 nM, 500 nM, 200 nM, 10 mM, 75 mM, and 10 μg/mL, respectively, the highest T_0 – T values were obtained. T_0 represents the threshold time for negative sample, and T denotes the threshold time for positive sample. [Target DNA] = 10 nM. Data are presented as mean values +/- standard deviation.

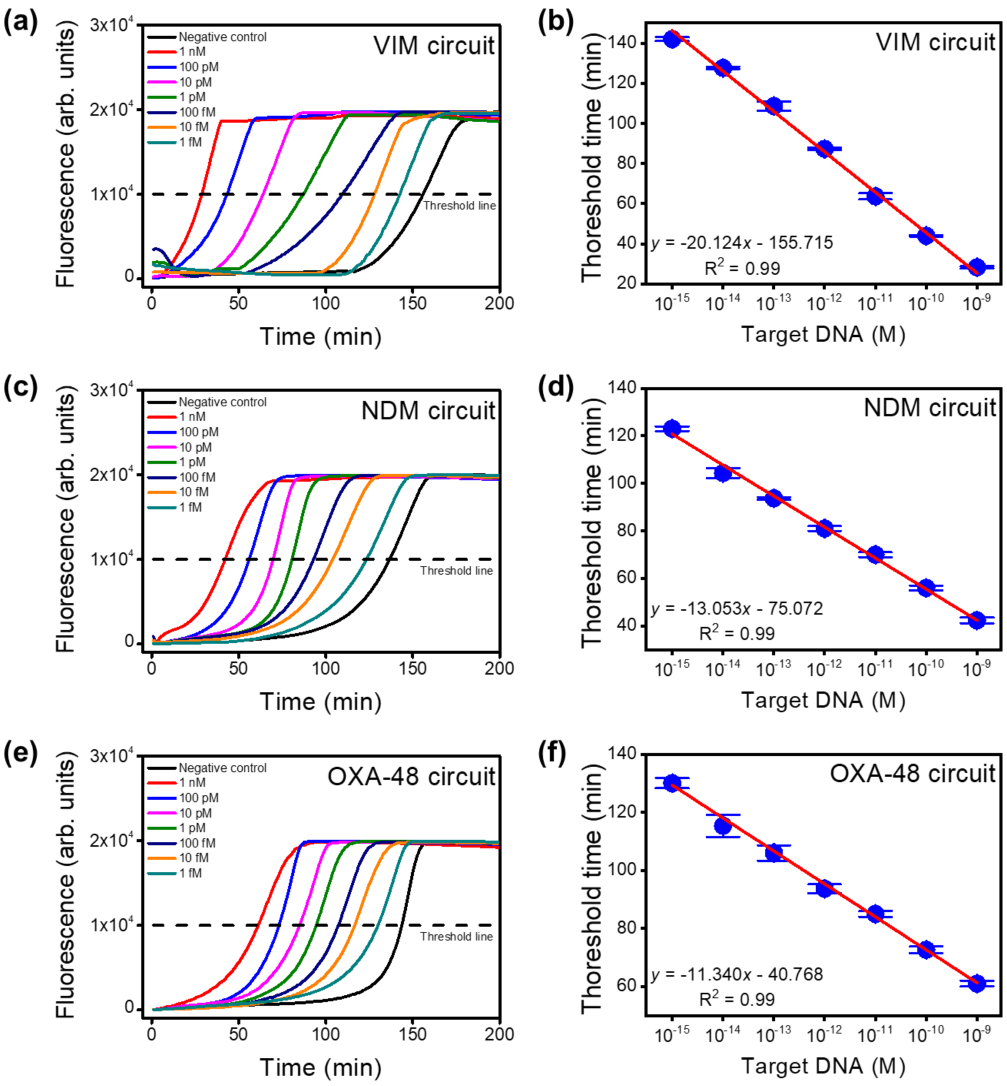

Supplementary Fig. 9 (a, c, e) Time-dependent fluorescence intensities during ANCA method with various concentrations of (a) VIM, (c) NDM, and (e) OXA-48 sequences. Dashed black threshold lines indicate the reaction time at which the fluorescence intensity reached 10,000 (threshold time). (b, d, f) Correlation of threshold time to the logarithm of (b) VIM, (d) NDM, and (f) OXA-48 concentration (n = 3 independent experiments, error bar = standard deviation). Red lines are linear fits, indicating LODs of 529 aM for VIM, 120 aM for NDM, and 144 aM for OXA-48. [R] = 500 nM, [R*] = 500 nM, [G1] = 25 nM, [G2] = 25 nM, [Ago] = 200 nM, [$MgCl_2$] = 10 mM, [NaCl] = 75 mM, and [BSA] = 10 µg/mL.

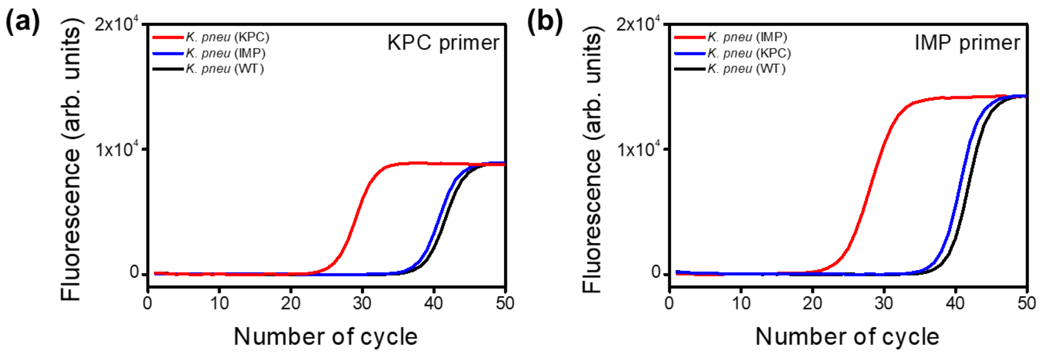

Supplementary Fig. 10 (a, b) RT-PCR results of bacteria using (a) KPC- and (b) IMP-specific primers. The experiments were independently replicated three times.

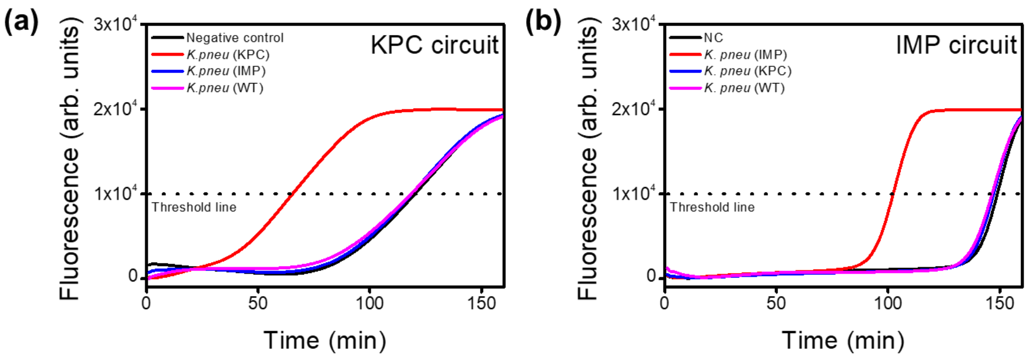

Supplementary Fig. 11 (a, b) Time-dependent fluorescence intensities corresponding to (a) Fig. 4b and (b) c. The experiments were independently replicated three times.

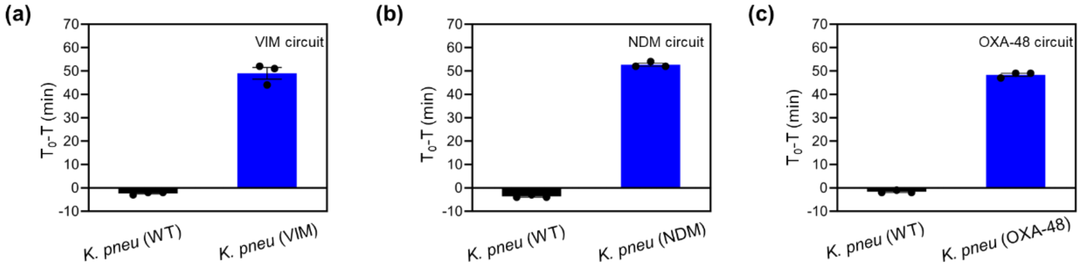

Supplementary Fig. 12 (a-c) Plot of T_0 – T value as a function of bacteria in buffer using (a) VIM, (b) NDM, and (c) OXA-48 circuits (n = 3 independent experiments, error bar = standard deviation). T_0 represents the threshold time for negative control sample (absence of bacteria), and T denotes the threshold time for test sample (presence of bacteria). Data are presented as mean values +/- standard deviation.

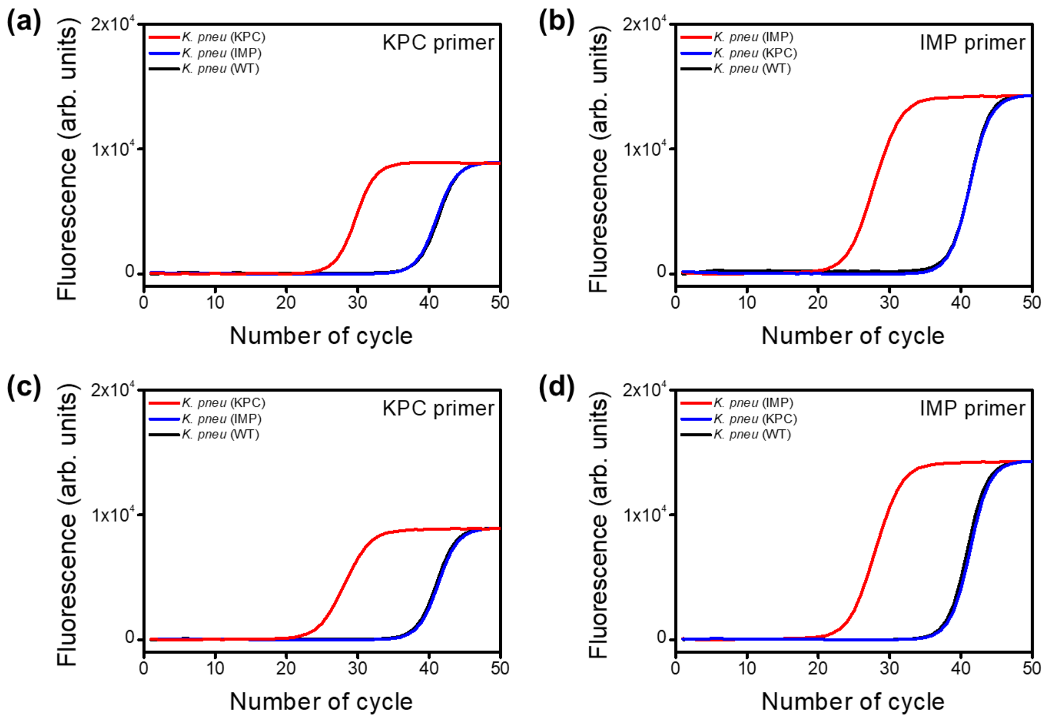

Supplementary Fig. 13 (a, b) RT-PCR results of bacteria in urine (99%) using (c) KPC- and (d) IMP-specific primer. (c, d) RT-PCR results of bacteria in blood (99%) using (e) KPC- and (f) IMP-specific primer. The experiments were independently replicated three times.

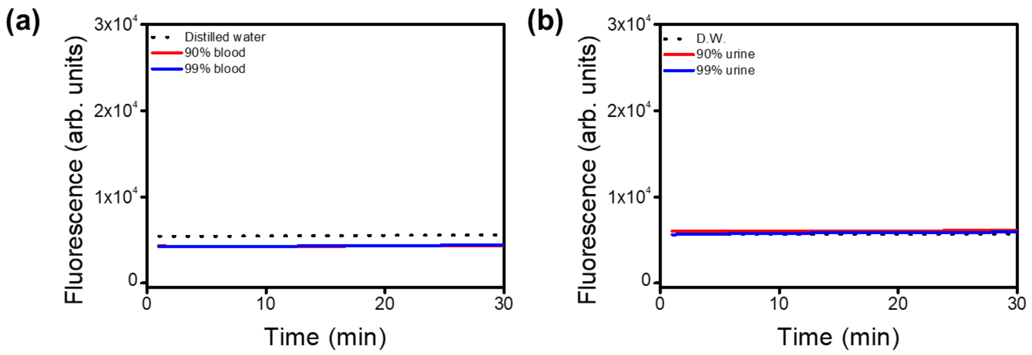

Supplementary Fig. 14 (a, b) Time-dependent fluorescence intensities from ANCA mixture with (a) urine and (b) blood samples. The experiments were independently replicated three times.

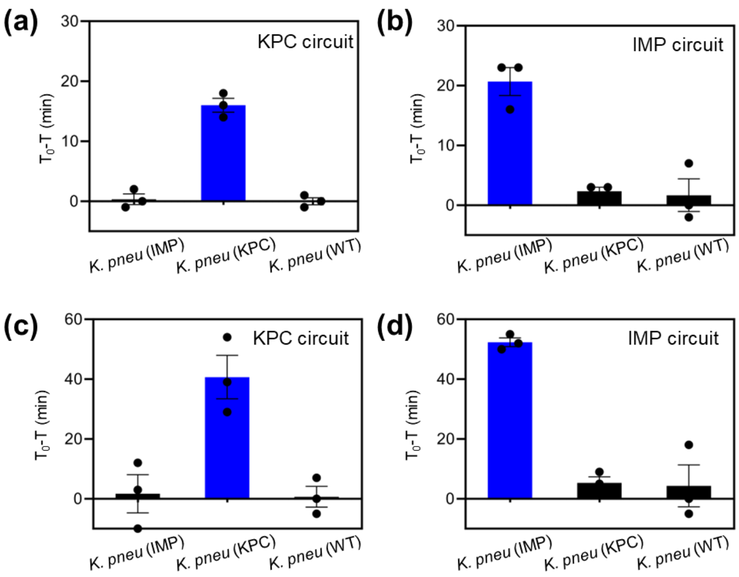

Supplementary Fig. 15 (a, b) Plot of T_0 – T value as a function of bacteria in urine (90%) using (a) KPC and (b) IMP circuits (n = 3 independent experiments, error bar = standard deviation). (c, d) Plot of T_0 – T value as a function of bacteria in blood (90%) using (c) KPC and (d) IMP circuits (n = 3 independent experiments, error bar = standard deviation). T_0 represents the threshold time for negative control sample (absence of bacteria), and T denotes the threshold time for test sample (presence of bacteria). Data are presented as mean values +/- standard deviation.

(a)

	KPC circuit		IMP circuit	
Sample	Negative	Positive	Negative	Positive
ANCA Positive	1	120	0	80
ANCA Negative	79	0	80	0
Agreement	NPA: 98.7%	PPA: 100%	NPA: 100%	PPA: 100%

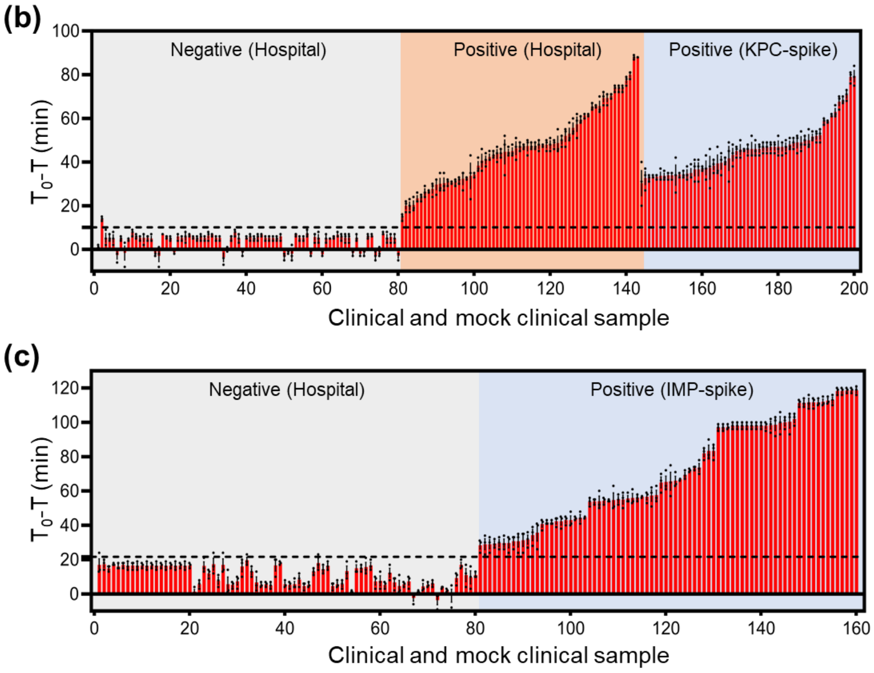

Supplementary Fig. 16 (a) Diagnostic results of ANCA method for clinical and CPKP-spiked samples (NPA: negative percentage agreement, PPA: positive percentage agreement). (b) Plot of $T_0 - T$ value as a function of clinical and *K. pneu* (KPC)-spiked sample (rectal swab-immersed transport media) (n = 3 independent experiments, error bar = standard deviation). (c) Plot of $T_0 - T$ value as a function of clinical and *K. pneu* (IMP)-spiked sample (rectal swab-immersed transport media) (n = 3 independent experiments, error bar = standard deviation). T_0 represents the threshold time for pure transport media, and T denotes the threshold time for clinical and mock clinical sample. Cut-off $T_0 - T$ values (10.11 and 21.61) are marked as dashed black lines. Data are presented as mean values +/- standard deviation.

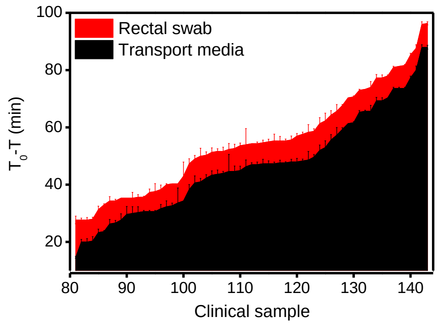

Supplementary Fig. 17 Comparative plot of $T_0 - T$ values obtained from rectal swab (red) and transport media (black) as a function of KPC-positive clinical samples (n = 3 independent experiments, error bar = standard deviation). Data are presented as mean values +/- standard deviation.

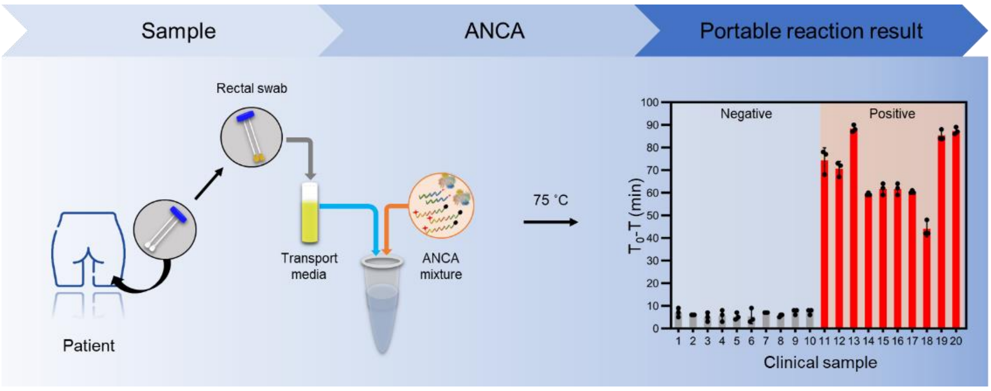

Supplementary Fig. 18 Schematic illustration for the diagnosis of CPKP from rectal swab-immersed transport media using ANCA portable isothermal nucleic acid amplification device and diagnostic results (n = 3 independent experiments, error bar = standard deviation). Data are presented as mean values +/- standard deviation.

Supplementary Table 1 Sequences of oligonucleotide used in this experiment.

Name	Sequence (5' → 3')	Target
KPC guide DNA 1 (G1)	P[(a)]-TATCA CTGTA TTGCACG	KPC
KPC guide DNA 2 (G2)	P[(a)]-CAAAT TGGCG GCGGCGT	
KPC Reporter (R)	GCGGCGT TATCACTGTA ACTAAAT	
KPC Reporter (R)-fluorophore modified	FAM-GCGGCGT TATCACTGTA ACTAAAT-BHQ2	
KPC Reporter complement (R*)	ATTTAGT TACAGTGATA ACGCCGC	
KPC Reporter complement (R*)-fluorophore modified	BHQ2-ATTTAGT TACAGTGATA ACGCCGC-FAM	
KPC trigger (T1)	TACAGTGATA ACGCCGC	
KPC forward primer	GGTTCTGTGGTCACCCATCT	
KPC reverse primer	TCCAGACGGAACGTGGTATC	
KPC synthetic target DNA	GGTGGCGGAGCTGTCCGCGGCCGCCGTGCAA TACAGTGATA ACGCCG CCGCCAATTTGTTGC	
KPC synthetic target complementary DNA	GCAACAAATTGGCGGCGGCGTTATCACTGTATTGCACGGCGGCCGCGGACAGCTCCGCCACC	
IMP guide DNA 1 (G1)	P[(a)]-TAACTTTTCA GTATCTT	IMP
IMP guide DNA 2 (G2)	P[(a)]-TCCACAAACC AAGTGAC	
IMP Reporter (R)	FAM-AAGTGAC TAACTTTTCA ACTAAAT-BHQ2	
IMP Reporter complement (R*)	ATTTAGT TGAAAAGTTA GTCACTT	
IMP trigger (T1)	TGAAAAGTTA GTCACTT	
IMP forward primer	CCTAAACATGGCTTGGTGGT	
IMP reverse primer	GCATACGTGGGGATAGATCG	
IMP synthetic target DNA	CTAATTGACACTCCATTTACGGCTAAAGATACTGAAAAGTTAGTCACTTGGTTTGTGGAGCGTG	
IMP synthetic target complementary DNA	CACGCTCCACAAACCAAGTGACTAACTTTTCAGTATCTTTAGCCGTAAATGGAGTGTCAATTAG	

Name	Sequence (5’ → 3’)	Target
VIM guide DNA 1 (G1)	P[(a)]-TAAACTACTA ATAACTT	VIM
VIM guide DNA 2 (G2)	P[(a)]-GCGGTCATGT AGACCAA	
VIM Reporter (R)	FAM-AGACCAA TAAACTACTA ACTAAAT-BHQ2	
VIM Reporter complement (R*)	ATTTAGT TAGTAGTTTA TTGGTCT	
VIM trigger (T1)	TAGTAGTTTA TTGGTCT	
VIM synthetic target DNA	ATGTTAAAAGTTAT TAGTAGTTTATTGGTCTACATGACCGCGTCTGTCATGGCTGTCGCAAGTC	
VIM synthetic target complementary DNA	GACTTGCGACAGCCATGACAGACGCGGTCATGTAGACCAATAAACTACTAATAACTTTTAACAT	
NDM guide DNA 1 (G1)	P[(a)]-TAAGTCGCAA TCCCCGC	NDM
NDM guide DNA 2 (G2)	P[(a)]-TCGACAACGC ATTGGCA	
NDM Reporter (R)	FAM-ATTGGCA TAAGTCGCAA ACTAAAT-BHQ2	
NDM Reporter complement (R*)	ATTTAGT TTGCGACTTA TGCCAAT	
NDM trigger (T1)	TTGCGACTTA TGCCAAT	
NDM synthetic target DNA	GGTATGGACGCGCTGCATGCGGCGGGGATTGCGACTTATGCCAATGCGTTGTCGAACCAGCTTG	
NDM synthetic target complementary DNA	CAAGCTGGTTCGACAACGCATTGGCATAAGTCGCAATCCCCGCCGCATGCAGCGCGTCCATACC	
OXA-48 guide DNA 1 (G1)	P[(a)]-TTTCTTAAAA AGCTGAT	OXA-48
OXA-48 guide DNA 2 (G2)	P[(a)]-ACTTATTGTG ATACAGC	
OXA-48 Reporter (R)	FAM-ATACAGC TTTCTTAAAA ACTAAAT-BHQ2	
OXA-48 Reporter complement (R*)	ATTTAGT TTTTAAGAAA GCTGTAT	
OXA-48 trigger (T1)	TTTTAAGAAA GCTGTAT	
OXA-48 synthetic target DNA	ATTCGAATTTCGGCCACGGAGCAAATCAGCTTTTTAAGAAAGCTGTATCACAATAAGT TACACG	
OXA-48 synthetic target complementary DNA	CGTGTAACTTATTGTGATACAGCTTTCTTAAAAAGCTGATTTGCTCCGTGGCCGAAATTCGAAT	

Name	Sequence (5' → 3')	Target
ureR forward primer	GGATATCTGACCAGTCGG	WT
ureR reverse primer	GGGTTTTGCGTAATGATCTG	

[(a)]P indicates the modification of phosphate group at 5'end.

Supplementary Table 2 Comparison of ANCA method with previous Ago-based nucleic acid detection methods.

Method	LOD	Target	Mechanism	Property	Ref
Ago-FISH (Argonaute-based fluorescence *in situ* hybridization)	1 pM	miRNA (Let7a, c)	Ago-based cleavage	- Low sensitivity	[S1]
MULAN (Multiplex Argonaute-based nucleic acid detection system)	1.6 copies/reaction	SARS-CoV-2 Influenza virus	RT-LAMP + Ago-based cleavage	- Requirement of reverse transcription and nucleic acid amplification steps (Multiple steps)<br>- Cannot be proceeded on isotherm	[S2]
NAVIGATER (Nucleic acid enrichment *via* DNA guided Argonaute from thermus thermophilus)	1 copy/reaction	KRAS mutation	PCR or RT-LAMP + Ago-based cleavage	- Requirement of nucleic acid amplification step (Multiple steps)<br>- Cannot be proceeded on isotherm	[S3]
A-Star (Ago-directed specific target enrichment and detection)	1 copy/reaction	BRAF & EGFR mutation	PCR + Ago-based cleavage	- Requirement of nucleic acid amplification step (Multiple steps)<br>- Cannot be proceeded on isotherm	[S4]
PLCR (Pfago coupled with modified ligase chain reaction for nucleic acid detection)	10 aM	SARS-CoV-2	Ligase chain reaction + Ago-based cleavage	- Requirement of reverse transcription and nucleic acid amplification steps (Multiple steps)<br>- Cannot be proceeded on isotherm	[S5]
PAND (Pfago-mediated nucleic acid detection)	1.6 aM	KRAS & EGFR mutation	PCR or tHDA + Ago-based cleavage	- Requirement of nucleic acid amplification step (Multiple steps)<br>- Cannot be proceeded on isotherm	[S6]
MAIDEN (Mesophilic Ago-based isothermal detection method)	4 nM	SARS-CoV-2	Reverse transcription + Ago-based cleavage	- Requirement of reverse transcription step (Multiple steps)<br>- Low sensitivity	[S7]
SPOT (Scalable and portable Testing)	0.44 copies/uL	SARS-CoV-2	RT-LAMP + Ago-based cleavage	- Requirement of reverse transcription and nucleic acid amplification steps (Multiple steps)<br>- Cannot be proceeded on isotherm	[S8]
TtAgoEAR (Ttago-based thermostable exponential amplification reaction)	10 aM	SARS-CoV-2	Ago-based cleavage + EXPAR	- Requirement of lots of proteins	[S9]

Method	LOD	Target	Mechanism	Property	Ref
NOTE-Ago (Novel and One-step cleavage method based on Argonaute by integrating Tag-specific primer extension and Exonuclease I)	1 CFU/mL	*S.* Typhi<br>*S. aureus*	PCR<br>+<br>Exo I-based digestion<br>+<br>Ago-based cleavage	- Requirement of nucleic acid amplification step (Multiple steps)<br>- Cannot be proceeded on isotherm	[S10]
ANCA	1.87 fM (KPC)<br>178 aM (IMP)<br>529 aM (VIM)<br>120 aM (NDM)<br>144 aM (OXA-48)	CPKP	Ago-based cleavage with nucleic acid circuit	- One-step<br>- Amplification-free<br>- Isothermal	This work

Supplementary Table 3 Comparison of ANCA method with previous CPE detection methods.

Method	Sensitivity	LOD	Advantage	Limitation	Ref
MHT (Culture-based Modified Hodge Test	>69%		- Simple and cost-effective	- Requires pure culture<br>- Long reaction time for culturing<br>- False-positive and false-negative	[S11]
CIM (Carbapenem-inactivation methods)	>90%		- Cover all carbapenemase<br>- Simple and cost-effective	- Requires pure culture<br>- Long reaction time for culturing	[S12]
Colorimetric assay based CarbaNP test and its automated kits	>70%		- Cover all carbapenemase<br>- Simple and cost-effective<br>- Low false-positive rate than MHT	- Hard to detect OXA-48 type producer<br>- Long reaction time for culturing	[S13]
MALDI-TOF	>92%		- Accurate data analysis<br>- High-throughput	- Requires expertise to analyze data<br>- Requirement expensive equipment<br>- Long reaction time for culturing	[S14]
LAMP	>90%		- Rapid and moderate cost<br>- Applicable in limited-resource settings	- Contamination due to amplification of target nucleic acid<br>- Complex primer design<br>- Requirement of DNA extraction	[S15]
Direct detection of rectal swabs by a multiplex lateral flow immunoassay	80-100%		- Simple and cost-effective<br>- Applicable in limited-resource settings	- Poor sensitivity<br>- Long reaction time for culturing	[S16]
SERS combined with chemometric tool	99.8%		- Rapid and moderate cost<br>- High sensitivity	- Poor reproducibility<br>- Requires specific instrument and multivariate data analysis	[S17]
DNA microarray	99.4%	10^3 copies/μL (~1.66 fM)	- High multiplexing<br>- High sensitivity	- Requirement of expensive equipment and DNA extraction<br>- Long reaction time for culturing	[S18]
RPA + CRISPR/Cas12	99.29%	4.48 fM	- High sensitivity and specificity<br>- Excellent accuracy	- Contamination due to amplification of target nucleic acid<br>- Multiple reaction steps	[S19]

Method	Sensitivity	LOD	Advantage	Limitation	Ref
LAMP + CRISPR/Cas12 + microfluidic chip		30 copies/reaction (~ 0.88 aM)	- On-site detection<br>- Naked eye detection<br>- No complicated device	- Multiple reaction steps<br>- Requirement of DNA extraction	[S20]
CRISPR/Cas12 + SWV		3.5 fM	- Excellent selectivity<br>- Simple and cost-effective	- Requirement of DNA extraction<br>- Multiple reaction steps	[S21]
ECL		0.05 copies/μL (~0.08 aM)	- High sensitivity<br>- Amplification-free	- Requirement of DNA extraction<br>- Multiple and long reaction steps	[S22]
ANCA	100%	1.87 fM (KPC)<br>178 aM (IMP)<br>529 aM (VIM)<br>120 aM (NDM)<br>144 aM (OXA-48)	- One-step<br>- Amplification-free<br>- Isothermal	- Ago-based cleavage with nucleic acid circuit	This work

Supplementary References

1. Shin, S. et al. Quantification of purified endogenous miRNAs with high sensitivity and specificity. *Nat. Commun.* **11**, 6033 (2020)
2. Ye, X. et al. Argonaute-integrated isothermal amplification for rapid, portable, multiplex detection of SARS-CoV-2 and influenza viruses. *Biosens. Bioelectron.* **207**, 114169 (2022)
3. Song, J. et al. Highly specific enrichment of rare nucleic acid fractions using Thermus thermophilus argonaute with applications in cancer diagnostics. *Nucleic Acids Res.* **48**, e19 (2020)
4. Liu, Q. et al. Argonaute integrated single-tube PCR system enables supersensitive detection of rare mutations. *Nucleic Acids Res.* **49**, e75 (2021)
5. Wang, L. et al. Pyrococcus furiosus Argonaute coupled with modified ligase chain reaction for detection of SARS-CoV-2 and HPV. *Talanta* **227**, 122154 (2021)
6. He, R. et al. Pyrococcus furiosus Argonaute-mediated nucleic acid detection. *Chem. Commun.* **55**, 13219-13222 (2019)
7. Li, X. et al. Mesophilic Argonaute-based isothermal detection of SARS-CoV-2. *Front. Microbiol.* **13**, 957977 (2022)
8. Wang, F. et al. PfAgo-based detection of SARS-CoV-2. *Biosens. Bioelectron.* **177**, 112932 (2021).
9. Yuan, C., Fang, J. & Fu, W. Thermus thermophilus Argonaute-Based Isothermal Amplification Assay for Ultrasensitive and Specific RNA Detection. *Anal. Chem.* **95**, 8291-8298 (2023).
10. Li, Y. et al. Argonaute-triggered visual and rebuilding-free foodborne pathogenic bacteria detection. *J. Hazard. Mater.* **454**, 131485 (2023).
11. Lutgring, J.D. & Limbago, B.M. The Problem of Carbapenemase-Producing-

Carbapenem-Resistant-Enterobacteriaceae Detection. *J. Clin. Microbiol.* **54**, 529-534 (2016).

12. Cui, X., Zhang, H. & Du, H. Carbapenemases in Enterobacteriaceae: Detection and Antimicrobial Therapy. *Front. Microbiol.* **10**, 1823 (2019).
13. Nordmann, P., Poirel, L. & Dortet, L. Rapid detection of carbapenemase-producing Enterobacteriaceae. *Emerg. Infect. Dis.* **18**, 1503-1507 (2012).
14. Gato, E. et al. Multicenter Performance Evaluation of MALDI-TOF MS for Rapid Detection of Carbapenemase Activity in Enterobacterales: The Future of Networking Data Analysis With Online Software. *Front. Microbiol.* **12**, 789731 (2021).
15. Nakano, R. et al. Rapid detection of the Klebsiella pneumoniae carbapenemase (KPC) gene by loop-mediated isothermal amplification (LAMP). *J. Infect. Chemother.* **21**, 202-206 (2015).
16. Vasilakopoulou, A., Karakosta, P., Vourli, S., Kalogeropoulou, E. & Pournaras, S. Detection of KPC, NDM and VIM-Producing Organisms Directly from Rectal Swabs by a Multiplex Lateral Flow Immunoassay. *Microorganisms* **9**, 942 (2021).
17. Bashir, S. et al. Rapid and sensitive discrimination among carbapenem resistant and susceptible E. coli strains using Surface Enhanced Raman Spectroscopy combined with chemometric tools. *Photodiagnosis Photodyn. Ther.* **34**, 102280 (2021).
18. Ma, X. et al. Development of a DNA microarray assay for rapid detection of fifteen bacterial pathogens in pneumonia. *BMC Microbiol.* **20**, 177 (2020).
19. Xu, H. et al. An Isothermal Method for Sensitive Detection of Mycobacterium tuberculosis Complex Using Clustered Regularly Interspaced Short Palindromic Repeats/Cas12a Cis and Trans Cleavage. *J. Mol. Diagn.* **22**, 1020-1029 (2020).
20. Wu, H. et al. A reversible valve-assisted chip coupling with integrated sample treatment and CRISPR/Cas12a for visual detection of Vibrio parahaemolyticus. *Biosens.*

Bioelectron. **188**, 113352 (2021).

21. Suea-Ngam, A., Howes, P.D. & deMello, A.J. An amplification-free ultra-sensitive electrochemical CRISPR/Cas biosensor for drug-resistant bacteria detection. *Chem. Sci.* **12**, 12733-12743 (2021).

22. Nikolaou, P. et al. Ultrasensitive PCR-Free detection of whole virus genome by electrochemiluminescence. *Biosens. Bioelectron.* **209**, 114165 (2022).
